# EXTRA-PANCREATIC MANIFESTATION IN AUTOIMMUNE PANCREATITIS VERSUS CONVENTIONAL PANCREATITIS: AN EGYPTIAN COHORT

**DOI:** 10.1590/S0004-2803.24612024-124

**Published:** 2025-10-27

**Authors:** Rania A M ABOUYOUSSEF, Mohamed Saied ABDELGAWAD, Mahmoud AGHA, Marwa IBRAHIM

**Affiliations:** 1 Tropical medicine department, Faculty of medicine, Alexandria university, Egypt.; 2 Diagnostic imaging department, National liver institute, Menoufia university, Egypt.; 3 Diagnostic imaging department, Medical research institute, Alexandria university, Egypt.

**Keywords:** AIP-Extrapancreatic-biliary-GB-renal-LNS-RPF, AIP-Extrapancreatic, biliar, GB-renal, LNS, RPF

## Abstract

**Background:**

Autoimmune pancreatitis (AIP) is a specific form of pancreatitis that is characterized by obstructive jaundice and sometimes associated with pancreatic masses, lymphoplasmacytic infiltrate and fibrosis, with a marked response to steroids. According to International Consensus Diagnostic Criteria AIP is categorized into type 1, type 2, and not otherwise specified (NOS). AIP-1 is one of the presentations of the IgG4-related disease (IgG4-RD) characterized by lymphoplasmacytic infiltration and more than ten IgG4-positive plasma cells per high-power field (HPF), storiform fibrosis, and obliterative phlebitis. Clinically, IgG4-RD is a systemic disease that can affect all organs. It can affect the bile ducts, kidneys, lymph nodes, prostate, and retroperitoneum.

**Objective:**

Recognize patients with extra pancreatic manifestation in autoimmune pancreatitis as soon as possible to achieve optimal outcomes.

**Methods:**

A retrospective comparative study was conducted on the previously hospitalized patients to our main university hospital, during the period from June 2022 till June 2024. It is a retrospective study that was done through revision of the patient clinical, laboratory and imaging including CT/MRI of the abdomen and chest CT of all patients. 60 patients with pancreatitis were enrolled in the study. It was diagnosed based on at least two criteria of the following: (1) typical abdominal pain, (2) elevated amylase and/or lipase greater than 3 times, and (3) radiological findings match with pancreatitis. Cases were divided according to the International Consensus Diagnostic Criteria (ICDC) into group A: Autoimmune pancreatitis (AIP) defined by a specific form of pancreatitis characterized by obstructive jaundice with or without pancreatic masses, lymphoplasmacytic infiltrate and fibrosis (from laparotomy biopsies in suspected pancreatic cancer) and a marked response to steroids. And group B: conventional pancreatitis, with 30 patients in each group.

**Results:**

A raised serum IgG4 was found in group A patients ranging from 135.0 mg/dL to 212.0 mg/dL with >95% specificity and sensitivity for AIP. The extra pancreatic associated diseases in AIP candidates, were detected in 23 patients (76.6%). Biliary tree complications were seen at 22 patients (73.3%), non calcular gall bladder disease was detected in 9 patients (30%), renal complications were found in 11 patients (36.6%), irrelevant lymphadenopathy in 10 patients (33.3%), retroperitoneal 3 patients (10%). In group B: there were biliary obstructive in 9 patients (30%), calcular cholecystitis was found in 19 patients (63%), with no other recorded extra pancreatic diseases. Chi square and Fisher Exact tests revealed significant differences between the two groups, as regards the extra pancreatic diseases in association with group A.

**Conclusion:**

Type 1 AIP has been associated with several extra pancreatic manifestations determining different clinical outcomes. Therefore, patient monitoring and a multisystem evaluation approach are required.

## HIGHLIGHTS

• Recognize patients with extra pancreatic manifestation in autoimmune pancreatitis.

• It is a retrospective study that was done through revision of the patient clinical, laboratory and imaging including CT/MRI of the abdomen and chest CT of all patients.

• The extra pancreatic associated diseases in AIP candidates, were detected in 23 patients (76.6%).

• Type 1 AIP has been associated with several extra pancreatic manifestations determining different clinical outcomes.

## RESUMO

**Contexto:**

A pancreatite autoimune (PAI) é uma forma específica de pancreatite que se caracteriza por icterícia obstrutiva e, por vezes, associada a massas pancreáticas, infiltrado linfoplasmocitário e fibrose, com resposta acentuada aos corticosteróides. De acordo com os Critérios de Diagnóstico de Consenso Internacional, a AIP é categorizada em tipo 1, tipo 2 e não especificada de outra forma (NOS). AIP-1 é uma das apresentações da doença relacionada à IgG4 (IgG4-RD) caracterizada por infiltração linfoplasmocitária e mais de dez plasmócitos IgG4-positivos por campo de grande aumento (HPF), fibrose estoriforme e flebite obliterativa. Clinicamente, a IgG4-RD é uma doença sistêmica que pode afetar todos os órgãos, incluindo os ductos biliares, rins, gânglios linfáticos, próstata e retroperitônio.

**Objetivo:**

Reconhecer pacientes com manifestação extra pancreática na pancreatite autoimune o mais rápido possível para alcançar os melhores resultados. Métodos: Foi realizado um estudo comparativo retrospectivo com pacientes previamente internados em nosso principal hospital universitário, durante o período de junho de 2022 a junho de 2024. Trata-se de um estudo retrospectivo que foi feito por meio de revisão clínica, laboratorial e de imagem do paciente, incluindo TC/RM do abdome e TC de tórax de todos os pacientes. Sessenta pacientes com pancreatite foram incluídos no estudo. Foi diagnosticada com base em pelo menos dois critérios: (1) dor abdominal típica, (2) amilase e/ou lipase elevada maior que 3 vezes e (3) achados radiológicos compatíveis com pancreatite. Os casos foram divididos de acordo com os Critérios Diagnósticos de Consenso Internacional (ICDC) no grupo A: pancreatite autoimune (AIP) definida, por uma forma específica de pancreatite caracterizada por icterícia obstrutiva com ou sem massas pancreáticas, infiltrado linfoplasmocitário e fibrose (de biópsias de laparotomia em suspeita de câncer de pâncreas) e uma resposta acentuada aos esteróides. E grupo B: pancreatite convencional, com 30 pacientes em cada grupo**.**

**Resultados:**

Um aumento de IgG4 sérica foi encontrado em pacientes do grupo A variando de 135,0 mg/dL a 212,0 mg/dL com especificidade de >95% e sensibilidade para AIP. As doenças associadas extrapancreáticas em candidatos a PAI, foram detectadas em 23 pacientes (76,6%). Complicações da árvore biliar foram observadas em 22 pacientes (73,3%), doença da vesícula biliar não calculada foi detectada em 9 pacientes (30%), complicações renais foram encontradas em 11 pacientes (36,6%), linfadenopatia irrelevante em 10 pacientes (33,3%), retroperitoneal 3 pacientes (10%). No grupo B: havia obstrução biliar em 9 pacientes (30%), calculando colecistite foi encontrada em 19 pacientes (63%), sem outras doenças extrapancreáticas registradas. Os testes qui-quadrado e Exato de Fisher revelaram diferenças significativas entre os dois grupos no que diz respeito às doenças extrapancreáticas em associação com o grupo A.

**Conclusão:**

A AIP tipo 1 tem sido associada a várias manifestações extrapancreáticas determinando diferentes desfechos clínicos. Portanto, o monitoramento do paciente e uma abordagem de avaliação multissistêmica são necessários.

## INTRODUCTION

Autoimmune pancreatitis (AIP) is considered a type of sterile chronic pancreatic disease; that is associated histologically with IgG4-positive plasma cells organ infiltration and tissue fibrosis. It can clinically present with abdominal pain, weight loss and obstructive jaundice. Imaging usually reveals a diffuse enlargement of the pancreas with or without a mass.

Autoimmune pancreatitis may lead to irreversible organ dysfunction if it is not managed promptly. Early steroid therapy is mandatory and could be considered as a therapeutic test. Steroid treatment is essential for reversing pancreatitis and for regaining normal pancreatic functions^1^.

AIP is an accepted term as its autoimmune characters are proven clinically, serologically, histologically, and by immunohistochemistry. AIP is sometimes associated with other autoimmune disorders such as Sjogren syndrome, idiopathic retroperitoneal fibrosis, and inflammatory bowel disease (IBD)^2^ it’s usually associated with hypergammaglobulinemia and increased serum levels of IgG, particularly IgG4^3^.

 AIP represents 5-6% of all patients with chronic pancreatitis, and 6-8% and 11% of resected pancreatic masses that was presumed preoperatively to be pancreatic cancer in Japan and Mayo clinic, respectively. However, the exact incidence is inaccurate because of the high number of underdiagnosed cases^4^.

AIP pathogenesis could be explained by genetic and immunologic mechanisms. Two distinct subtypes were identified: type 1 and type 2 AIP. Type1 AIP, or lymphoplasmacytic sclerosing pancreatitis (LPSP), has abundant perivascular plasma cells infiltrates (IgG4 cells) together with lymphocytes infiltration and fibrosis. This perivascular infiltration ends in obliterative phlebitis, which is the major histopathological feature of type 1 AIP^5^.

Type 2 AIP has another histopathological pattern, named idiopathic duct-centric pancreatitis (IDCP) or granulocytic epithelial lesion (GEL). AIP type 2 is associated with destruction and obliteration of the pancreatic duct, with no serological elevation of IgG4 or the presence of autoantibodies, or other extra pancreatic organ involvement. Inflammatory bowel disease was the only autoimmune disease linked with AIP type 2 (approximately 30%)^6^ ^,^ ^7^.

AIP type I is suggested by some authors to be just a part of a systemic pathological process, that may involve other extra-pancreatic organ involvement. Few published articles were found discussing the possible involvement of the gall bladder, biliary ducts, kidneys, thyroid, salivary and lacrimal glands, orbits, lymphatic system, lungs, gastrointestinal tract, and blood vessels and mesentery^8^ ^,^ ^9^.

On the contrary, acute usual pancreatitis is an acute inflammatory disease, with various degrees of severity. It may be present with different severity grades according to Balthazar grading and necrosis percentage. It may also be associated with some extra-pancreatic diseases, as causative or aggravating factors or as a complication. Taking this conventional pancreatitis as a control group of this study is helpful for attaining accurate sensitivity and specificity indices.

The aim of the present work was to recognize patients with extra pancreatic manifestation in autoimmune pancreatitis as soon as possible to achieve optimal outcomes.

## METHODS

A single-center retrospective comparative study was conducted upon two groups. Group A consisted of 30 patients with AIP type 1, while group B included 30 patients with conventional pancreatitis, with different severity degrees. The study was done through revision of clinical, laboratory and imaging including - computerized tomography (CT)scan chest and abdomen of all patients and magnetic resonance imaging (MRI) / magnetic resonance cholangiopancreatography (MRCP) in some patients.

### Exclusion criteria

Any patient with known comorbidities, e.g. (diabetes mellitus, hypertension, chronic kidney disease, chronic lung disease, cardiac disease, cancer, collagenic, neurological diseases) was excluded. Complete clinical and neurological examinations and laboratory investigations were done.

### Candidates’ inclusion criteria

Group A (AIP): 

1- Positive imaging findings.

2- High amylase level.

3- lymphoplasmacytic infiltrate and fibrosis.

4 - Positive response to steroid therapy.

Group (B): usual pancreatitis.

1- Positive imaging findings.

2- High amylase level.

A CT scan of chest and abdomen (multidetector computed tomography (MDCT) tailored for pancreatic imaging including non-contrast, pancreatic, portal and delayed phases.) was done for initial diagnosis of AIP, as well as for any suspected associated different organ pathology.

MRI examination including MRCP, using a 1.5 T closed MRI imager (Avanto, Siemens, Erlangen, Germany) was done to several patients. The pulse sequences used were transverse T2FSE with and without fat saturation, T1 chemical shift sequences (In/opposed phase), Dynamic pre- and post-gadolinium VIBE sequences, and MRCP sequences (Thin slice 3D, as well as thick slab single shot).

### Ethical approval

Approval of the local Ethics Committee of University of Alexandria was obtained.

### Statistics

Data was analyzed using IBM SPSS software package version 20.0. (Armonk, NY: IBM Corp). The Kolmogorov- Smirnov was used to verify the normality of distribution of variables, Comparisons between groups for categorical variables were assessed using chi-square test (Fisher Exact). Significance of the obtained results was judged at the 5% level.

## RESULTS

In this study the age ranged from 16 to 62 years, with an average of 32 years in both groups.

The commonest initial clinical manifestation was obstructive jaundice in 60-70% of patients followed by abdominal pain or abdominal discomfort. mild epigastric pain was also found in about one third of patients with autoimmune pancreatitis. Other clinical manifestations included weight loss, abnormalities of exocrine and endocrine pancreatic functions.

In group B abdominal pain and acute pancreatitis were more frequent. Acute pancreatitis occurred in nearly 50% of patients. Other manifestations included painless obstructive jaundice and focal pancreatic masses.

A raised serum IgG4 was found in group A patients ranging from 135.0 mg/dL to 212.0 mg/dL with >95% specificity and sensitivity for AIP.

### Extra pancreatic associated diseases

In group A: biliary tree complications were detected in patients 23 (76.6%), non calcular gall bladder disease in patients 18 (60%), calcular cholecystitis in patients 4 (13.3%), renal complications in patients 11 (36.6%), irrelevant lymphadenopathy in 10 patients (33.3%) and retroperitoneal fibrosis in 3 patients (10%). At group B: there were biliary obstructive disease in patients 9 (30%), calcular cholecystitis in patients 19 (63%), with no other recorded extra pancreatic diseases.

### Comparative statistic between group A and group B with the use of Chi square and Fisher Exact tests

Biliary complications: *P* value 0.034, sensitivity 76.6%, specificity 70%, gall bladder diseases: *P* value 0.045, sensitivity 60%, specificity 16.6%, renal diseases: *P* value 0.001, sensitivity 34.67%, specificity 100.0%, Mediastinal Lymph nodes: *P* value 0.001, sensitivity 33.33%, specificity 100.0%, retroperitoneal fibrosis (RPF): *P* value 0.237, sensitivity 10% and specificity 100.0% (Table 1).

**TABLE 1:** Comparison between the two groups according to different parameters.

	Group A (n=30)	Group B (n=30)	c^2^	*P*	Sensitivity	Specificity
Biliary	23 (76.7%)	9 (30%)	13.125^*^	0.034^*^	76.67	70.0
GB	18 (60%)	25 (83.3%)	4.022^*^	0.045^*^	60.0	16.67
Renal	11 (36.6%)	0 (0%)	12.954^*^	0.001^*^	34.67	100.0
Mediastinal LNs	10 (33.3%)	0 (0%)	12.0^*^	0.001^*^	33.33	100.0
RPF	3 (10%)	0 (0%)	3.158	*P*=0.237^FE^	10.0	100.0

c^2^: chi square test; FE: Fisher Exact; *P*: *P* value for comparing between the two studied groups; *Statistically significant at *P*≤0.05.

## DISCUSSION

AIP is considered a part of the new clinicopathologic entity entitled IgG4-related autoimmune diseases. AIP is frequently associated with various extra pancreatic lesions, these lesions associated with AIP have pathologic features typical of those found in AIP. Although Serum IgG4 levels are often high this is not a disease-specific finding^10^.

AIP is diagnosed Radiologically using a CT scan; there is a diffuse or segmental enlargement of the pancreas with effaced or lost pancreatic clefts, with a sausage-shaped pancreas. In contrast to the usual pancreatitis, AIP usually shows no peri-pancreatic fat planes stranding.

AIP is diagnosed mostly by a biopsy in patients undergoing pancreaticoduodenectomy for presumed pancreatic cancer. Despite the growing awareness of this condition, differentiating AIP and pancreatic cancer remains a challenging matter, especially for patients with radiologic evidence of a tumefactive lesion^11^ ^,^ ^12^.

Although AIP can result in various extra pancreatic lesions like primary sclerosing cholangitis, this can be differentiated by IgG4+ plasma cell infiltration detected around the pancreatic ducts as well as adequate response to corticosteroid therapy. Due to AIP association with extra-pancreatic manifestations and other autoimmune diseases, autoimmune pancreatitis is sometimes referred to as a systemic illness^13^.

### Biliary complications

In Group A - 23 (76.6%) patients were recorded to have different combinations of sclerosing cholangitis (SC) as well as pancreatic ducts complications. Variable degrees of diffuse mural thickening of the biliary ducts were found, multisegmented beaded intra-and extrahepatic biliary dilatation, distal CBD smooth tapering stricture was also detected.

This is in consistent with Nagpal SJS et al., who stated that AIP-1 usually occurs with the involvement of other organs, such as biliary stricture, renal involvement, orbital pseudotumor, extensive lymphadenopathy, and retroperitoneal fibrosis^14^.

Several case series have also described the association between AIP and biliary strictures. The overall rate of extrahepatic bile duct involvement in AIP is 71% to 100%^15^ ^-^ ^17^.

**FIGURE 1:**
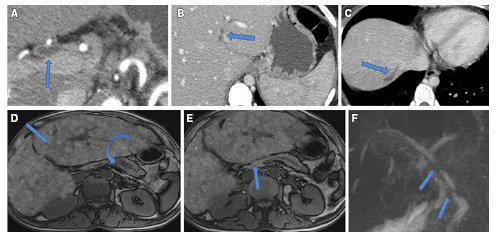
AIP triphasic CT scan (A) arterial (B, C&D) Portal phases: showing thickened wall slightly dilated CHD (Arrow at A), beaded IHBD (Arrow at B), isolated subcapsular IHBD (Arrow at C). Another AIP patient MRI: (D) Biliary cirrhosis (Arrow) with cystic distal pancreatic duct dilatation (Curved arrow), (E) Proximal pancreatic duct stricture (Arrow)& (F) MRCP showing multisegmented CBD stricture. (Arrow).

Primary biliary cirrhosis (PBC) was detected in only two patients (6.6%) (Figure 1). These associated biliary abnormalities could be explained by the immune basis of this AIP disease. Considering the systemic nature of IgG 4 AIP, it may suggest that the coexistence of AIP, Sclerosing cholangitis and PBC is not a rare coincidence, but sometimes coexist as due to shared genetic susceptibility, as well as the coexistence of plasma cells infiltration of specimens of these diseases^18^ ^-^ ^20^.

In group B: there were 9 (30%) cases of biliary complications, seen as common bile duct (CBD) dilatation with different degrees of intrahepatic biliary dilatation. Only of them 2 (22.2%) had CBD stones, with considerable CBD dilatation >12 mm and Intra hepatic biliary dilatation. The other patients 7 (88.8%) had no CBD stones, with 8 mm average CBD caliber. Non calcular biliary ducts dilatation, associated with acute conventional pancreatitis, could be explained by the presence of viscid biliary secretions. This leads to cholestasis, with secondary CBD dilatation^21^ ^,^ ^22^.

### Gall bladder

**FIGURE 2:**
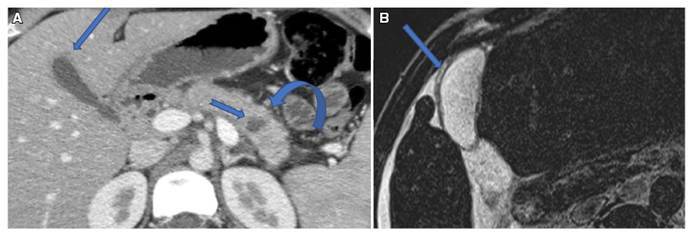
AIP (A) CT scan showing GB wall thickening (arrow), cystic pancreatic duct dilatation (Notched arrow), Halo sign (curved arrow) (B): AIP with associated circumferential gall bladder wall thickening. (Arrow)

In Group A - A considerable gall bladder diffuse wall thickening was detected without associated stones, i.e. non calcular cholecystitis like pattern was detected in 9 patients. (30%). It occurs due to diffuse wall infiltration with IgG4-positive plasma cells and transmural fibrosis. The gallbladder wall appears thickened, hypoechoic in ultrasound and hypodense at early arterial and portal phases of CT scan, with delayed enhancement at the equilibrium delayed phases^23^ (Figure 2).

This was also reported by Kamisawa T et al., in his study, where severe or moderate thickening of the gallbladder wall was radiologically detected in 10 of 19 (53%) AIP patients; in these 10 patients, stenosis of the extrahepatic bile duct was also observed^24^.

**FIGURE 3:**
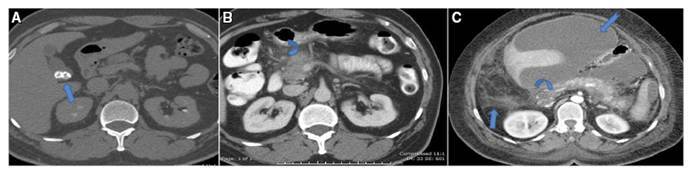
(A-B) Acute conventional pancreatitis: (A) gall stones, (B) extensive peripancreatic fat planes stranding and small lymphadenopathy (curved arrow). (C) Another patient with extensive pancreatic necrosis (Curved arrow), fat planes standing (Arrow) and massive collection (Notched Arrow).

In group B: 19 patients (63%) were seen to have gall bladder stones and calcular cholecystitis. However, calcular cholecystitis is the cause of group B pancreatitis, not a complication. This could be explained by the migration of small stones through the CBD, to be lodged at the ampulla of Vater. This triggers a local activation of pancreatic enzymes leading to auto pancreatic digestion, that causes different severity degrees of acute pancreatitis^25^ (Figure 3).

Gallbladder is frequently affected in AIP; a diffuse, acalculous, lymphoplasmacytic cholecystitis is usually found, this pattern is highly specific for extrahepatic biliary tract disease, but it does not differentiate between primary and secondary cholangiopathies such as PSC, malignant obstructive jaundice, or gall stones^26^ ^,^ ^27^.

### Renal involvement

In this study, we recorded renal complications in association with AIP in 11 patients (36.6%). These renal insults had been seen in two patterns; 8 patients (26.6%) had interstitial nephritis, while only three patients (10%) had cortical nodules. All renal insults were associated with different amounts of perinephric fluid collection (FIGURE 2).

Interstitial nephritis was seen as diffuse renal enlargement, with alternating hyper-and hypodense linear strands of enhancing and non-enhancing tissues, parallel to the axis of the tubules. This gives the kidneys the typical striated nephrogram, at the nephrographic phase. This matches some recent articles, which were published by Merkle M^28^, who reported interstitial nephritis as associated extra pancreatic complications of AIP.

The other 3 patients showed cortical nodules, in a round or wedge-shaped configuration. These nodules were seen as hypodense cortical spots at the nephrographic phase, with some mild enhancement on delayed phases. These nodules could be explained by the localized lymphoplasmacytic infiltration. In association with perinephric collection, this cortical affection may simulate pyelonephritis, especially since most of these nodules were ill defined. However, sterile perinephric fluid tapping, and clear urine analysis were supportive findings of the immune mediated associated renal insults of these patients.

Only 4 of these 11 renal patients (36.3%) developed transient compromise of renal functions, with elevated serum creatinine for 1 month averaging period. None of our patients developed permanent renal failure or hydronephrosis. This matches the findings described by Vlachou P et al. in Radiographic 2011 published study^29^.

Also, NakamurinH, stated that renal lesions in Interstitial nephritis usually present as tubulointerstitial nephritis. Patients can present clinically with a deranged renal function, vasculitis, or a renal mass; these symptoms often are associated with AIP. Histologically, renal lesions show a densely patchy or diffuse tubulointerstitial lymphoplasmacytic infiltrate. Numerous eosinophils are often seen, Tubulitis and tubular injury are also present, along with tubular atrophy and focal thickening of the tubular basement membranes. Glomeruli are usually not involved^30^.

**FIGURE 4:**
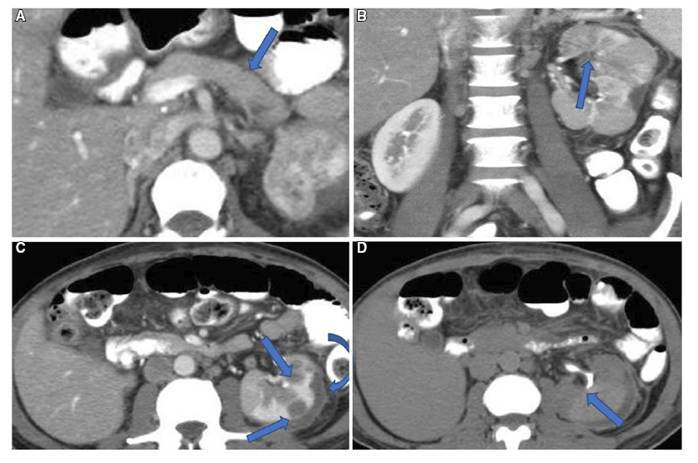
AIP with left renal complications: A- Sausage shaped pancreas with hypodense rim (Arrow), (B) left renal enlargement with striated nephogram (Arrow) (C) Multiple cortical nodules (Arrows) and perinephric collection (Curved arrows) (D) preserved renal function with good excretion (Arrow).

These AIP associated renal findings support the concept of IgG4-related systemic disease proposed in the related published literatures. So, IgG4-related autoimmune disease could be included in the pathogenesis of tubulointerstitial nephritis. One should keep in mind that, if there is an unexplained combination of pancreatic and renal inflammatory processes IgG4-related autoimmune disease is an explanation. All these candidates with renal involvement associated with AIP, were followed up for 6 months, showing complete recovery, with no permanent renal damage^31^ (Figure 4).

### Lymphadenopathy

**FIGURE 5:**
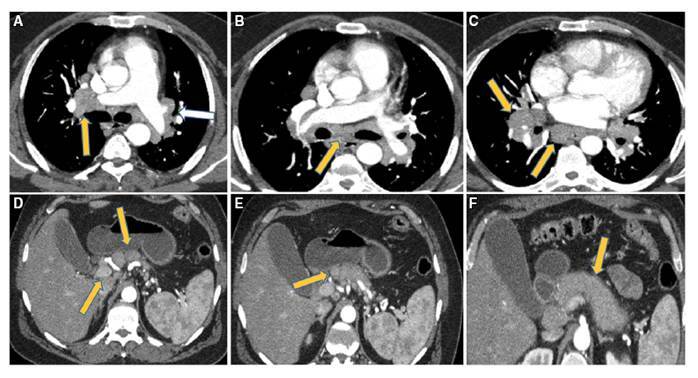
Lower chest and upper abdominal scans revealing: extensive sizable bilateral hilar, and subcarinal lymphadenopathy (Arrows A-C), celiac, superior mesenteric and peripancreatic lymphadenopathy (Arrows D&E), sausage shaped diffusely enlarged, normally enhancing pancreas with hypodense rim (AIP) (Arrows F).

Irrelevant distant lymphadenopathy was recorded in 10 patients (30%) in group A. Lymphadenopathy was considered, if there were more than one group of lymph nodes affected and nodal short axis distance more than 15 mm. This was seen at mediastinal, hilar, cervical, mesenteric, iliac and paraaortic groups. This could be explained by the condensed infiltration of lymph nodes with IgG4-positive plasma cells which is pathologically proven. All these patients with lymphadenopathy showed good response to steroids; with noticeable resolution in short time averaging 3 weeks^32^ (Figure 5).

This is in accordance with Uchida K et al., who described lymphadenopathy as a well-known extra pancreatic lesion in type 1 autoimmune pancreatitis (AIP) that appears synchronously or metachronously with type 1 AIP. He also stated that IgG4-related lymphadenopathy may sometimes mimic or be misdiagnosed as lymphoma^33^.

### Retroperitoneal fibrosis (RPF)

**FIGURE 6:**
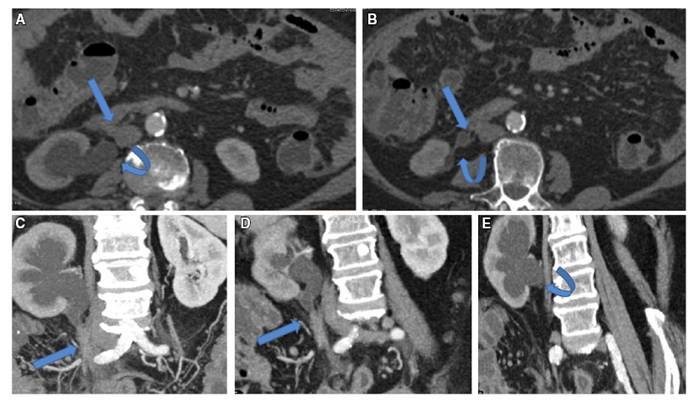
CT (A&B) axial, (C-E) Coronal MPR showing: AIP patient showing right retroperitoneal fibrotic band (Arrows), entrapping and compromising the upper right ureter, with secondary right upper hydroureter and hydronephrosis (Curved arrows).

Although uncommon, there are reported articles and published thesis about the association between AIP and RFP, whatever synchronous or metachronous. In our study, we recoded only three cases (10%) of synchronous RFP and AIP (Figure 6). It was right sided retroperitoneal ill-defined fibrous tissue bands, which encased and compromised the right upper ureter. It induced short segmental upper hydroureter and grade II hydronephrosis.

Choi et al., also found in his retrospective study that out of 52 patients with idiopathic retroperitoneal fibrosis 27 cases were IgG4-related. He also found other organ involvement was much more frequent in IgG4-related RPF (50% vs 0%), and the pancreas was the most affected organ^34^.

This could be explained by the pathological analysis of specimens in some previously published articles. These retroperitoneal masses or bands were periureteral fibrosis with abundant infiltration of IgG4-positive plasma cells and lymphocytes and obliterative phlebitis. 

These reports confidently stated a common pathophysiological mechanism for retroperitoneal fibrosis and autoimmune pancreatitis in these tested cases^35^ ^-^ ^38^.

Some cases of retroperitoneal fibrosis associated with AIP have been reported and were resolved by corticosteroid therapy, also serum IgG4 levels were elevated for most patients^39^.

## CONCLUSION

There is no doubt that autoimmune pancreatitis is still a challenging diagnosis. It should be considered and kept in mind, that it is usually associated with other organs and systems affection. It is important to include chest and neck in the imaging evaluation of AIP, to avoid initially missing the diagnosis and thus possibly delayed complications.

## References

[1] Hamano H, Kawa S, Horiuchi A, Unno H, Furuya N, Akamatsu T (2001). High serum IgG4 concentrations in patients with sclerosing pancreatitis. N Engl J Med.

[2] Fukukura Y, Fujiyoshi F, Nakamura F, Hamada H, Nakajo M (2003). Autoimmune pancreatitis associated with idiopathic retroperitoneal fibrosis. AJR Am J Roentgenol.

[3] Hirano K, Kawabe T, Yamamoto N, Nakai Y, Sasahira N, Tsujino T (2006). Serum IgG4 concentrations in pancreatic and biliary diseases. Clin Chim Acta.

[4] Chari ST, Smyrk TC, Levy MJ, Topazian MD, Takahashi N, Zhang L (2006). Diagnosis of autoimmune pancreatitis: the Mayo Clinic experience. Clin Gastroenterol Hepatol.

[5] Okazaki K, Uchida K, Sumimoto K (2014). Autoimmune pancreatitis: pathogenesis, Autoimmune pancreatitis: pathogenesis, latest developments and clinical guidance. Ther Adv Chronic Dis.

[6] Chari S, Zhang G, Notohara K, Lerch M, Shimosegawa T (2010). Histopathologic and clinical subtypes of autoimmune pancreatitis: the honolulu consensus document. Pancreas.

[7] Klöppel G, Detlefsen S, Chari S (2010). Autoimmune pancreatitis: The clinicopathological characteristics of the subtype with granulocytic epithelial lesions. J Gastroenterol.

[8] Manfredi R, Frulloni L, Mantovani W, Bonatti M, Graziani R, Mucelli R (2011). Autoimmune pancreatitis: pancreatic and extrapancreatic MR imaging-MR cholangiopancreatography findings at diagnosis, after steroid therapy, and at recurrence. Radiology.

[9] Bodily K, Takahashi N, Fletcher JG (2009). Autoimmune pancreatitis: pancreatic and extrapancreatic imaging findings. AJR Am J Roentgenol.

[10] Hamano H, Arakura N, Muraki T, Ozaki Y, Kiyosawa K, Kawa S (2006). Prevalence and distribution of extrapancreatic lesions complicating autoimmune pancreatitis. J Gastroenterol.

[11] Hardacre JM, Iacobuzio-Donahue CA, Sohn TA, Abraham SC, Yeo CJ, Lillemoe KD (2003). Results of pancreaticoduodenectomy for lymphoplasmacytic sclerosing pancreatitis. Ann Surg.

[12] Weber SM, Cubukcu-Dimopulo O, Palesty JA, Suriawinata A, Klimstra D, Brennan MF (2003). Lymphoplasmacytic sclerosing pancreatitis: inflammatory mimic of pancreatic carcinoma. J Gastrointest Surg.

[13] Kamisawa T, Tu Y, Nakajima H, Egawa N, Tsuruta K, Okamoto A (2006). Usefulness of biopsying the major duodenal papilla to diagnose autoimmune pancreatitis: a prospective study using IgG4-immunostaining. World J Gastroenterol.

[14] Nagpal SJS, Sharma A, Chari ST (2018). Autoimmune Pancreatitis. Am J Gastroenterol.

[15] Ohara H, Nakazawa T, Sano H, Ando T, Okamoto T, Takada H (2005). Systemic extrapancreatic lesions associated with autoimmune pancreatitis. Pancreas.

[16] Chari ST, Smyrk TC, Levy MJ, Topazian MD, Takahashi N, Zhang L (2006). Diagnosis of Autoimmune Pancreatitis: The Mayo Clinic Experience. Clin Gastroenterol Hepatol.

[17] Kamisawa T, Nakajima H, Egawa N, Funata N, Tsuruta K, Okamoto A (2006). IgG4-related sclerosing disease incorporating sclerosing pancreatitis, cholangitis, sialadenitis and retroperitoneal fibrosis with lymphadenopathy. Pancreatology.

[18] Bia Y, Hartae P, Lawa R (2016). Obstructive jaundice in autoimmune pancreatitis can be safely treated with corticosteroids alone without biliary stenting. Pancreatology.

[19] Nishino T, Toki F, Oyama H (2005). Biliary tract involvement in autoimmune pancreatitis. Pancreas.

[20] Kawa S, Ota M, Yoshizawa K (2002). HLA DRB10405-DQB10401 haplotype is associated with autoimmune pancreatitis in the Japanese population. Gastroenterology.

[21] Frossard J, Steer ML, Pastor C (2008). Acute pancreatitis. Lancet.

[22] Malik A (2015). Acute pancreatitis. A more common and severe complication of gallstones in males. Int J Health Sci.

[23] Yamabe A (2017). Inflammatory Bile Duct Obstruction during the Healing Stage of Severe Acute Pancreatitis. Intern Med.

[24] Kamisawa T, Tu Y, Nakajima H, Egawa N, Tsuruta K, Okamoto A (2006). Sclerosing cholecystitis associated with autoimmune pancreatitis. World J Gastroenterol.

[25] Hitoshi Y, Tu Y, Nakajima H (2006). Sclerosing cholecystitis associated with autoimmune pancreatitis. World Journal of Gastroenterology.

[26] Kamisawa T, Tu Y, Nakajima H, Egawa N, Tsuruta K, Okamoto A (2006). Sclerosing cholecystitis associated with autoimmune pancreatitis. World J Gastroenterol.

[27] Abraham SC, Cruz-Correa M, Argani P, Furth EE, Hruban RH, Boitnott JK (2003). Lymphoplasmacytic chronic cholecystitis and biliary tract disease in patients with lymphoplasmacytic sclerosing pancreatitis. Am J Surg Pathol.

[28] Merkle M, Gröne M (2012). Interstitial nephritis and autoimmune pancreatitis: a case report. Int Urol Nephrol.

[29] Vlachou P, Khalili K, Jang H (2011). IgG4-related Sclerosing Disease: Autoimmune Pancreatitis and Extrapancreatic Manifestations. RadioGraphics.

[30] Nakamura H, Wada H, Origuchi T, Kawakami A, Taura N, Aramaki T (2006). A case of IgG4-related autoimmune disease with multiple organ involvement. Scand J Rheumatol.

[31] Takahashi N, Kawashima A, Fletcher JG, Chari ST (2007). Renal involvement in patients with autoimmune pancreatitis: CT and MR imaging. Radiology.

[32] Alidjan F, Karim F, Verdijk R (2015). A Patient with Autoimmune Pancreatitis Type 1 with Previously Known Lymphadenopathy, Both in the Context of IgG4-related Disease. Am J Case Rep.

[33] Uchida K, Okazaki K, Kamisawa T, Chung J (2015). Autoimmune Pancreatitis.

[34] Choi YK, Yang JH, Ahn SY, Ko GJ, Oh SW, Kim MG (2019). Retroperitoneal fibrosis in the era of immunoglobulin G4-related disease. Kidney Res Clin Pract.

[35] Tan T, Ng Y, Tan D (2014). Extrapancreatic findings of IgG4-related disease. Clin Radiol.

[36] Papadopoulos G, Skandalakis P, Filippou D (2018). Autoimmune pancreatitis associated with retroperitoneal fibrosis mimicking cholangiocarcinoma. Oxford Medical Case Reports.

[37] Kamisawa T, Chen PY, Tu Y, Nakajima H, Egawa N (2006). Autoimmune pancreatitis metachronously associated with retroperitoneal fibrosis with IgG4-positive plasma cell infiltration. World J Gastroenterol.

[38] Ohtsubo K, Watanabe H, Tsuchiyama T, Mouri H, Yamaguchi Y, Motoo Y (2007). A case of autoimmune pancreatitis associated with retroperitoneal fibrosis. Jop.

[39] Kim J, Byun J, Lee S, Kim H, Lee M (2013). Atypical Manifestations of IgG4-Related Sclerosing Disease in the Abdomen: Imaging Findings and Pathologic Correlations. AJR.

